# Acute suppurative thyroiditis in a 13-year-old boy: a rare pediatric case report

**DOI:** 10.1097/RC9.0000000000000553

**Published:** 2026-05-26

**Authors:** Abdulrahman Mohammed Abdulrahman Abouh, Esra Altayeb Alhady Massry, Ahmed Idris Abdelrahman Idris, Farah Hamid Yousif Dawood, Mohammedbabalrahma Bashier Ahmed Koko, El Bushra Ahmed Doumi

**Affiliations:** a Department of General Surgery, Faculty of Medicine & Health Sciences, University of Kordofan, El Obeid, Sudan; b Department of Surgery, El Obeid Teaching Hospital, El Obeid, Sudan; c Surgery Department, Sheikan College, El Obeid, North Kordofan State, Sudan; d Department of Surgery, Faculty of Medicine and Health Sciences, University of Kordofan, El Obeid, Sudan

**Keywords:** hemithyroidectomy, neck infection, pediatric, thyroid abscess

## Abstract

**Introduction and importance:**

Thyroid abscess is an uncommon condition in children due to the gland’s natural resistance to infection, provided by its encapsulation, vascularity, lymphatic drainage, and iodine content. Delay in diagnosis may result in serious complications such as airway compromise and mediastinitis. Early recognition and appropriate intervention are essential to prevent morbidity.

**Case presentation:**

A pediatric boy presented with a 2-month history of anterior neck swelling that had rapidly increased in size over the preceding 2 weeks, associated with 1 week of fever. Examination showed a tender, warm, oval swelling over the left thyroid lobe that moved with swallowing, along with ipsilateral cervical lymphadenopathy. Laboratory studies revealed mild leukocytosis with neutrophilia and elevated C-reactive protein, while thyroid function tests were normal. Neck ultrasound demonstrated a phlegmonous inflammatory process with a small intrathyroidal fluid collection; computed tomography (CT) was unavailable. Fine-needle aspiration cytology indicated pyogenic thyroiditis, and culture grew *Staphylococcus aureus*. He underwent left hemithyroidectomy with drainage of necrotic tissue and pus. His postoperative recovery was uneventful, and he was discharged in good condition.

**Clinical discussion:**

Pediatric thyroid abscess is rare and often mimics other neck pathologies, contributing to delayed presentation. In resource-limited settings, ultrasound and fine needle aspiration cytology provide adequate diagnostic guidance when CT is unavailable. Surgical intervention remains the mainstay of treatment, especially in cases of significant necrosis or well-formed abscesses. This case highlights the effectiveness of timely surgical management in achieving excellent outcomes.

**Conclusion:**

Thyroid abscess should be considered in children with progressive neck swelling and fever. Early diagnosis and timely surgical management are vital to prevent complications and ensure favorable outcomes.

## Introduction

The thyroid abscess is a rare condition that is most commonly experienced by immunocompromised patients, those with anatomical abnormalities, or those who already have thyroid problems^[^1^]^. Approximately 0.7%–1% of all thyroid patients suffer from thyroid abscess or acute suppurative thyroiditis; however, thyroid abscess alone is extremely rare, occurring in about 0.1%–0.7% of all thyroid cases. As a result of its well-enveloped capsule, abundant blood supply, and high iodine content, the thyroid gland is normally resistant to infection^[^2,3^]^.

HIGHLIGHTS

- Thyroid abscesses are extremely rare in children due to the gland’s natural resistance to infection.
- This 13-year-old boy presented with progressive anterior neck swelling and fever, which was confirmed as a thyroid abscess.
- Management required a hemithyroidectomy with drainage, followed by an uneventful recovery.
- The case emphasizes diagnostic challenges and successful management in a resource-limited setting without computed tomography imaging.

A thyroid gland abscess should be considered by physicians as a differential diagnosis in patients who experience rapid onset anterior neck swelling in order to make an early diagnosis and institute proper management^[^4^]^. In spite of the fact that thyroid abscesses are uncommon, they can result in significant morbidity and mortality. Therefore, when a thyroid abscess is diagnosed, aggressive management should be initiated to prevent dangerous complications. Most of the thyroid abscesses are successfully managed with a combination of antibiotics and surgery^[^5^]^.

We present a rare case of bacterial thyroid abscess in a 13-year-old boy caused by *Staphylococcus aureus* and successfully managed with hemithyroidectomy. This manuscript was prepared following the SCARE guidelines^[^6^]^.

## Case presentation

A 13-year-old boy presented with a 2-month history of anterior neck swelling, which had progressively increased in size over the preceding 2 weeks. This was associated with fever and chills for 7 days, as well as decreased oral intake with odynophagia over the last 3 days. There was no history of heat intolerance, diarrhea, tremors, convulsions, or focal neurological deficits, and no history of similar episodes in the past. Additionally, there was no history suggestive of trauma, recurrent infections, or underlying immunodeficiency.

**Figure 1.**
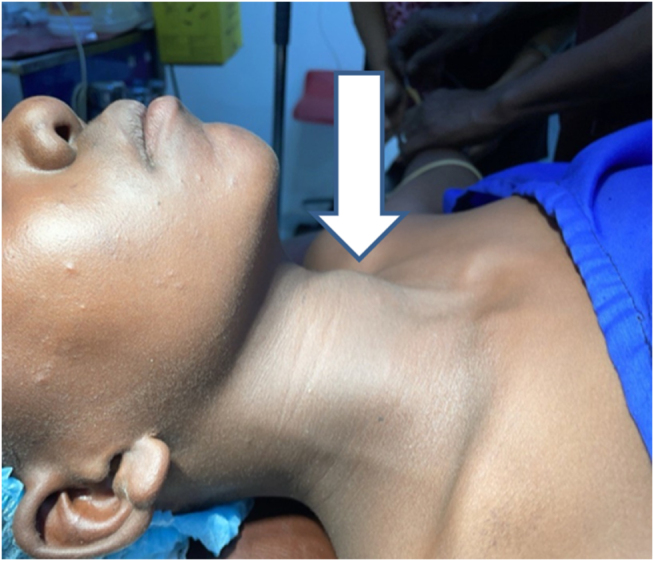
Patient preoperative with visible swelling in the left side of anterior triangle of the neck.

On examination, the patient was febrile at admission, with other vital parameters within normal limits, and the systemic examination was unremarkable. The patient had a left-sided swelling in the anterior aspect of the neck, measuring approximately 4 × 3 cm (Fig. 1). The skin over the swelling was normal. The swelling moved with swallowing but not with protrusion of the tongue. On palpation, the swelling was soft and tender, with a mildly increased local temperature. There was palpable cervical lymphadenopathy. Oral cavity and oropharyngeal examinations were normal.

Laboratory investigations showed normal thyroid function tests. Full blood count revealed mild leukocytosis with neutrophilia. C-reactive protein was elevated at 35.5 mg/L, and the erythrocyte sedimentation rate was 65 mm/h. Renal function tests, electrolytes, and liver function tests were within normal limits, and the chest X-ray was clear.

Neck ultrasound showed a heterogeneous mass involving the left thyroid lobe, measuring approximately 7 × 6 × 4 cm, with internal thick fluid consistent with an abscess, along with cervical lymphadenopathy. Fine-needle aspiration cytology revealed pyogenic thyroiditis with growth of *S. aureus* and no evidence of tuberculosis. Computed tomography (CT) was not performed due to unavailability in our setting; however, ultrasound combined with fine needle aspiration cytology (FNAC) was sufficient to confirm the diagnosis and guide surgical management.

**Figure 2.**
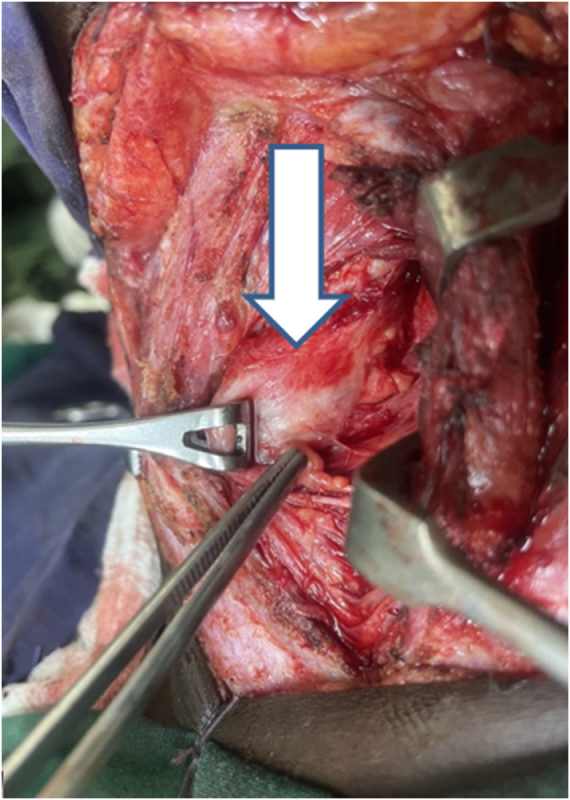
Intraoperative photograph showing left thyroid lobe with thick capsule with pus.

The patient was started on empirical intravenous antibiotics (ceftriaxone and metronidazole) with supportive management. He subsequently underwent a left hemithyroidectomy through a transverse cervical incision. Intraoperatively, necrotic thyroid tissue and purulent material were identified and drained. The diseased lobe was excised with preservation of the recurrent laryngeal nerve and parathyroid glands. A drain was placed, and the wound was closed in layers. The procedure was uneventful (Fig. 2).

**Figure 3.**
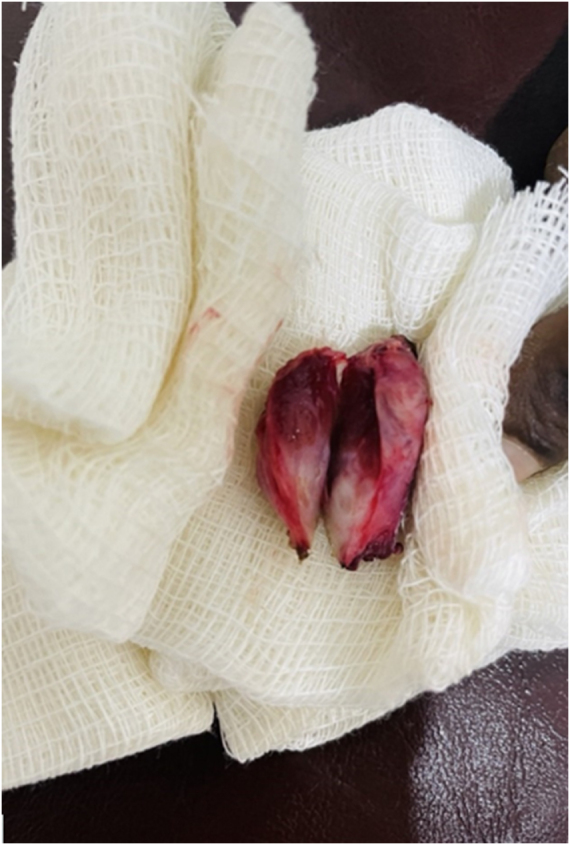
Postoperative photograph showing left thyroid lobe with thick capsule and necrosis tissues.

The patient had an uneventful postoperative recovery and was discharged on postoperative day 4 with oral antibiotics, along with a scheduled follow-up at 2 weeks and 1 month. Culture results confirmed the presence of *S. aureus* (Fig. 3).

## Discussion

Thyroid abscess in children is a very rare thyroid disorder, accounting for less than 1% of thyroid diseases^[^7,8^]^. Most cases are associated with congenital anomalies, particularly pyriform sinus fistula, which may predispose to recurrent infections^[^2,9,10^]^. Other predisposing factors include trauma, immunodeficiency, and systemic infection. In our patient, no history of trauma, recurrent infections, or clinical features suggestive of immunodeficiency was identified, and routine laboratory evaluation did not indicate an underlying immunocompromised state^[^1,11,12^]^.

Common symptoms include tender neck swelling, fever, dysphagia, and occasionally airway obstruction^[^12–14^]^. In this patient, the swelling progressed gradually, with fever onset in the final week. Cervical lymphadenopathy can mimic other infections or neoplastic processes. Differential diagnoses considered included thyroglossal duct cyst, cervical lymphadenitis, and other congenital neck masses; however, these were excluded based on clinical findings (absence of movement with tongue protrusion), imaging characteristics, and cytological results^[^5,15^]^.

Laboratory studies often show leukocytosis, neutrophilia, and elevated inflammatory markers, while thyroid function is generally normal^[^10,16,17^]^. Imaging, including ultrasound and CT scan, is crucial for identifying abscesses, delineating extent, and guiding surgical intervention^[^1,2,14,18,19^]^. FNAC confirms pyogenic thyroiditis and helps exclude tuberculosis or malignancy. In this case, FNAC was particularly important to confirm the diagnosis, identify the causative organism, and exclude alternative diagnoses such as tuberculosis and malignancy^[^4,15^]^.

Although a CT scan is useful in defining the extension and underlying causes, such as a pyriform sinus fistula, it was not available in our setting. Nevertheless, ultrasound combined with FNAC was adequate to establish the diagnosis and guide appropriate treatment. This highlights the value of a pragmatic diagnostic approach in resource-limited settings where advanced imaging is not readily accessible.

Common pathogens include *S. aureus, Streptococcus* species, and, rarely, *Salmonella* or fungal organisms^[^13,14,17^]^. Pediatric cases can occasionally involve mucormycosis, particularly in immunocompromised children^[^8,13^]^.

Management involves intravenous antibiotics and abscess drainage; percutaneous aspiration may suffice in selected cases^[^7,15,20^]^, while hemithyroidectomy is indicated for extensive necrosis, recurrent abscesses, or suspected congenital anomalies. Compared with previously reported cases managed conservatively or with aspiration alone, surgical intervention in our case was justified due to the presence of necrotic tissue and a well-formed abscess, ensuring complete source control and preventing recurrence^[^12,16,20^]^.

In this patient, hemithyroidectomy with drainage achieved excellent results.

With timely diagnosis and management, outcomes are generally favorable^[^17,19^]^. Delayed intervention may lead to airway compromise or systemic infection^[^8,13^]^.

## Conclusion

Thyroid abscess in children is rare but potentially serious. Clinicians should maintain a high index of suspicion in children with progressive neck swelling and fever. Early imaging, cytology, and timely surgical intervention ensure optimal outcomes.

## Data Availability

All data generated during this study are included in this puplished article.

## Research registration unique identifying number (UIN)

1. Name of the registry: Research Registry. 2. Unique identifying number or registration ID: researchregistry11684. 3. Hyperlink to your specific registration (must be publicly accessible and will be checked): https://www.researchregistry.com/browse-the-registry#home.
